# An Evolutionary Game Model with Punishment and Protection to Promote Trust in the Sharing Economy

**DOI:** 10.1038/s41598-019-55384-4

**Published:** 2019-12-24

**Authors:** Manuel Chica, Raymond Chiong, Marc T. P. Adam, Timm Teubner

**Affiliations:** 1 Andalusian Research Institute DaSCI “Data Science and Computational Intelligence”, University of Granada, 18071 Granada, Spain; 2 School of Electrical Engineering and Computing, The University of Newcastle, Callaghan, NSW 2308 Australia; 3 Einstein Center Digital Future, TU Berlin, 10587 Berlin, Germany

**Keywords:** Computational science, Cultural evolution

## Abstract

In this paper, we present an evolutionary trust game, taking punishment and protection into consideration, to investigate the formation of trust in the so-called sharing economy from a population perspective. This sharing economy trust model comprises four types of players: a trustworthy provider, an untrustworthy provider, a trustworthy consumer, and an untrustworthy consumer. Punishment in the form of penalty for untrustworthy providers and protection in the form of insurance for consumers are mechanisms adopted to prevent untrustworthy behaviour. Through comprehensive simulation experiments, we evaluate dynamics of the population for different initial population setups and effects of having penalty and insurance in place. Our results show that each player type influences the ‘existence’ and ‘survival’ of other types of players, and untrustworthy players do not necessarily dominate the population even when the temptation to defect (i.e., to be untrustworthy) is high. Additionally, we observe that imposing a heavier penalty or having insurance for all consumers (trustworthy and untrustworthy) can be counterproductive for promoting trustworthiness in the population and increasing the global net wealth. Our findings have important implications for understanding trust in the context of the sharing economy, and for clarifying the usefulness of protection policies within it.

## Introduction

Many forms of social and economic transactions are built on the decision maker’s expectation that their transaction partner will not exploit their vulnerability and behave opportunistically by deviating from previously made agreements. This expectation is commonly conceptualised as *trust*, that is, the “belief that the other party will behave in a dependable, ethical, and socially appropriate manner” (^1^, p. 53). The belief that another party will behave trustworthily is particularly important for Internet-facilitated transactions, which are characterised by high levels of uncertainty about the other party’s intention and limited enforceability compared to offline channels^2^. Trust, in this regard, can be considered as the willingness to make oneself vulnerable to the actions of another party^3^.

In recent years, there has been a rapid proliferation of new platforms that facilitate consumer-to-consumer (C2C) transactions between private individuals. In this so-called *sharing* or *platform economy*, consumers share access to private resources such as accommodation (Airbnb, Homestay), cars (Turo, Drivy), or rides (BlaBlaCar) with other users. In contrast to the more traditional form of C2C commerce, such as selling commodities on eBay, transactions in this sharing economy do not involve transfer of ownership. Instead, there are new opportunities for private individuals to (1) act as providers and monetise their private assets in multiple transactions without loss of ownership, and (2) act as consumers and get access to a broader range of resources, potentially at lower prices than by conventional modes of consumption. Beyond such economic motives, the literature has identified several aspects that drive individual engagement in sharing economy transactions, including social and sustainability reasons^4–6^. However, any of these motives will only translate into actions if there is a sufficient level of trust between the transaction partners.

In contrast to traditional e-commerce, such sharing transactions are commonly carried out in the provider’s private sphere (e.g., their home or car), often entailing personal interactions and a high level of economic exposure as they come with the risk of theft or damage to private assets. To this end, the realisation of a transaction does not only require the consumer’s belief that the *provider* will behave trustworthily, but also the provider’s belief that the *consumer* will behave trustworthily. At the nexus of this mutual trusting constellation, emerging research aims to provide insights into how trust can be established in such platform ecosystems, typically building on surveys^6,7^ and laboratory experiments^8,9^. What is common to these studies, though, is that they focus on the perspectives of individual users. These studies also do not capture the potential insights that could be gained through studying the evolution of trust and trustworthiness in the broader context of the ecosystem as a whole.

To bridge this gap, we use evolutionary game theory (EGT) as a framework and propose a sharing economy trust model with *punishment* and *protection* in place. Within the EGT framework, social dilemma models such as the Prisoner’s Dilemma and Snowdrift games have been adopted to study cooperative phenomena extensively^10,11^. In contrast, limited studies have used EGT to model trust^12–15^. In our model, players can either be *providers* or *consumers*. Providers and consumers can choose to be trustworthy or untrustworthy, thereby giving rise to four possible strategies: being a *trustworthy provider* (*TP*), an *untrustworthy provider* (*UP*), a *trustworthy consumer* (*TC*), and an *untrustworthy consumer* (*UC*). Punishment in the form of penalties for untrustworthy providers and protection in the form of insurance for consumers are mechanisms adopted to promote trust in the sharing economy environment.

We opt for agent-based modelling^16,17^ over a purely analytical approach to model the game and its players’ interactions. Agent-based modelling is a computational approach, representing individuals in the population with agents (or players). Each of them is given an opportunity to act autonomously based on a set of rules. The agents/players can be located either in a well-mixed environment or on a social network. Network structures restrict the game’s interactions to local neighbourhoods^18,19^. Update rules (e.g., proportional imitation^20^) are used to evolve players’ strategies depending on payoffs obtained in previous simulation steps.

Through extensive computational experiments comparing the level of trust and net wealth under different initial population conditions and parameters of the model, we show that trust can be formed when rewards for trustworthy consumers and providers are high, except if the initial population has a limited number of trustworthy players. Surprisingly, untrustworthy consumers are almost never ‘dominant’, because they are ‘vulnerable’ to untrustworthy providers. Instead, trustworthy providers and consumers drive net wealth by ‘cooperating’ with each other. Our experimental results also show that for most parameter configurations, having heavy penalties for untrustworthy providers does not increase net wealth; sometimes a heavier penalty can even decrease trustworthiness. Finally, insurance for consumers can be effective in promoting trust, but it should be limited to trustworthy consumers only.

## Background and Motivation

In the past decades, EGT has been used extensively as a standard framework for understanding the emergence and maintenance of cooperation. Based on the development and study of different social dilemma models, significant advancements have been made, not only in evolutionary biology, but also in other fields such as anthropology, computer science, economics, operations research, physics, political and social sciences, and psychology, among others (see Chapter 1 of Sandholm’s book^21^ for historical EGT developments). Two of the most commonly studied models of cooperation are the Prisoner’s Dilemma^22^ and Snowdrift^23^ games.

### Evolutionary trust games

Different from models of cooperation and simultaneous decision scenarios, the trust game is sequential in nature. In a classical trust game, there are two types of players: an investor and a trustee^24^. The investor *first* decides whether and to which degree to put faith in the trustee (e.g., by transferring some amount of money), and *then* the trustee decides whether to return the investment (i.e., to act trustworthily) or not.

Trust games have gained attention from the EGT community relatively recently, where McNamara *et al*.^25^ showed that allowing investors to obtain information about trustees’ past behaviour at a cost can promote trust and trustworthiness. Manapat *et al*.^14^ considered a scenario where information about trustees is not always available, and found that it can alter the nature of game interaction, leading to the evolution of fully trusting and marginally trustworthy behaviour. Tarnita^15^ explored non-random interactions in a structured environment using the classical evolutionary trust game, and showed that the population structure biases selection towards strategies that are both trusting and trustworthy.

Abbass *et al*.^12^ introduced an *N*-player trust game, and demonstrated that, in a well-mixed environment, even with just a single untrustworthy player in the initial population, untrustworthiness would spread very quickly leading to the extinction of investors. However, they also noticed that a fraction of the population would always remain trustworthy, even in the absence of investors. Chica *et al*.^13^ extended the *N*-player trust game by examining the effects of having different social network topologies. They found that trust can be promoted if players are connected via a social network, and more interestingly, they showed that the heterogeneity of a network topology influences trust evolution depending on the level of difficulty of the game. Subsequently, they also studied the effects of different update rules based on the extended *N*-player trust game^26^.

Clearly, only a limited number of studies related to evolutionary trust games can be found in the relevant literature. Following our initial attempt^27^ in studying evolutionary dynamics of four possible strategies in the sharing economy context, here we incorporate punishment and protection into our model to see if trust can be further promoted.

### Punishment and insurance

Even though typical sharing economy transactions hinge on sufficient levels of mutual trust and good conduct, the involved players are not always fully at their interaction partner’s mercy. In particular, platform operators employ a variety of mechanisms or tools to (a) protect users from indecent and mischievous behaviour by installing means of *punishment* and (b) mitigate the potential risks and detrimental effects of damage by means of *insurance*.

For instance, most platforms allow their users to denounce (and hence punish) misbehaviour. The most common of such features are functions to report fraudulent user profiles, numerical star rating scores, and text reviews^28,29^. Note that on most sharing platforms, the overall distribution of rating scores is skewed towards highly positive ratings^30^. Negative reviews for providers will thus draw attention and hence have greater impact. Bad ratings, in fact, represent a great threat to any platform user’s aspiration to future transactions and are therefore a powerful means to ensure compliance and good conduct. Moreover, providers with positive scores are able to attract more demand and to impose higher prices for their goods and services in the market^31–33^. Taxi service platform Uber operates an even more direct punishment scheme, by which drivers whose ratings drop below 4.6 (out of 5.0) stars are at risk of being banned from the platform^34^.

One key impediment to sharing is users’ concern about harm due to transaction partners’ unobservable actions, yielding a moral hazard^35^. Despite platforms’ considerable efforts to prevent wrongdoing, peer-based transactions are, by their very nature, vulnerable to crime, miscommunication, poor service quality, and interpersonal conflict. In order to protect customers in such cases, many platforms have installed insurance mechanisms that help, relocate, or reimburse affected customers. Airbnb’s policy, for instance, protects customers against accommodation listings that are unclean, unsafe, not adequately accessible, or misrepresented online by offering a refund or assisting with alternative accommodation^36^. Similarly, the former ride sharing platform Carpooling (now BlaBlaCar) used to offer compensatory train tickets for stranded passengers, for instance, if a ride was canceled on a short notice or when the driver did not show up^37^. As such, insurance may contribute to facilitating the initialisation and realisation of transactions, not by building trust but by mitigating potential consequences and hence lowering the required trust thresholds. Other (quasi-insurance) measures include fiduciary payment services and the promotion of codes of conduct (such as Airbnb’s non-discrimination appeal).

## Methods

### Game definitions and payoffs

Our sharing economy trust model consists of a finite set of agents occupying the nodes of a real network, and the edges denote interactions or ‘transactions’ between them (both for accumulating payoffs and strategy updating^38^). Transactions in the sharing economy are facilitated by matching individuals who have mutual interest in sharing resources, typically through an online network. We use a social network, defined by a set of actors/agents and the relationships (connections) among them, to represent this online network. The intuition for this modelling is that each agent has a relationship with (1) providers who can provide the resources they need and (2) consumers who demand assets that the agents can provide them. In other words, the network reflects a matching of complementary provider-consumer relationships of private individuals rather than a social relationship in the traditional sense.

All agents in the population play the game over a fixed number of time steps. Each agent *i* can choose one of the four possible strategies at every time step $s(i)=\{TP,UP,TC,UC\}$:

- *TP*: A trustworthy provider who offers an asset as promised;
- *UP*: An untrustworthy provider who offers an asset with markedly lower levels of product/service quality compared to what was promised beforehand;
- *TC*: A trustworthy consumer who uses an asset facilitated by a provider in a dependable, socially and economically appropriate manner;
- *UC*: An untrustworthy consumer who uses an asset facilitated by a provider in an excessive, socially or economically inappropriate manner (e.g., by damaging or stealing it, molesting others, etc.).

The game is based on pairwise interactions^39^. That is, every agent interacts or ‘transacts’ with other directly connected agents in pairs. The initial population of size *Z* is generated at random with the above-mentioned four strategies. The net wealth of individual agents, calculated based on their payoffs, is determined according to the strategy adopted by themselves and those they interact with. The total net wealth, *w*_*i*_, of focal agent *i* is then calculated by adding the payoff values of all its interactions with other agents. See Table 1 for the payoff matrix.

**Table 1:** Payoffs for the sharing economy trust game.

Providers	Consumers	
	*TC*	*UC*
*TP*	*R*, *R*	−S, *Temp*
*UP*	$\mathrm{(1}-p)X$, $({d}_{T}-\mathrm{1)}X$	$\mathrm{(1}-p)X$, $({d}_{U}-\mathrm{1)}X$

Note: *Temp* is the temptation for a consumer to be untrustworthy towards a trustworthy provider; *R* is the reward when both the provider and consumer are trustworthy; *S* is the sucker punishment for a trustworthy provider when a consumer is untrustworthy; and *X* is the value an untrustworthy provider keeps and a consumer–trustworthy or untrustworthy–pays after a transaction is initiated (like a deposit). Here, *Temp*, *R*, *S* and *X* can be any values greater or equal to zero.

The model’s payoffs also include a penalty when providers are untrustworthy (*UP*), noted by *p* ∈ [0, 1), and insurance for consumers when they make a transaction with an untrustworthy provider. The insurance is applicable to both *TC* and *UC*, defined as *d*_*T*_, *d*_*U*_ ∈ [0, 1), respectively. Note that when both players interacting with each other are of the same role (i.e., both are consumers or both are providers), no payoff is given.

The payoff matrix in Table 1, which measures the cost and gain for the different types of players during the game, is designed based on the fact that the provider-consumer interaction in typical sharing economy scenarios constitutes a social dilemma situation, where both players have an incentive to deviate from the initial agreement, hence requiring substantial levels of mutual trust^9^. In this context, taking on a trustworthy role represents the cooperative strategy, whereas taking on the untrustworthy role represents the strategy of defection. There exists a Pareto-optimal (i.e., no players’ payoffs can be increased without decreasing the payoff of at least one other player) but unstable constellation of mutual cooperation, in which both the provider and the consumer receive payoffs of *R*. In this situation, both parties execute their sharing transaction as per previously agreed-upon conditions. However, if a consumer decides to defect against a trustworthy provider, a *Temp* payoff is given to the untrustworthy consumer at a cost, −*S*, to the trustworthy provider. If providers act in an untrustworthy manner, a payoff of *X* is given to them while the consumers are penalised with a payoff of −*X*. This means untrustworthy players are able to exploit their counterparts’ good faith economically, hence giving untrustworthy strategies the edge over trustworthy strategies.

We denote the numbers of trustworthy providers, untrustworthy providers, trustworthy consumers, and untrustworthy consumers in a local neighbourhood (excluding the focal agent itself) with *k*_*TP*_, *k*_*UP*_, *k*_*TC*_, and *k*_*UC*_, respectively. The equality ${k}_{TP}+{k}_{UP}+{k}_{TC}+{k}_{UC}={\langle k\rangle }_{i}$ must always be fulfilled for consistency’s sake, where ${\langle k\rangle }_{i}$ is the degree (number of connections) of a focal agent. By taking this notation and the payoff matrix into account, we can define the net wealth *w*_*i*_ of focal agent *i* as follows:

1 $${w}_{i}=\{\begin{array}{ll}{k}_{TC}R-{k}_{UC}S, & if\,s(i)=TP\,({\rm{trustworthy}}\,{\rm{provider}})\,,\\ ({k}_{TC}+{k}_{UC})(1-p)X, & ifs\,(i)=UP\,({\rm{untrustworthy}}\,{\rm{provider}}),\\ {k}_{TP}R+{k}_{UP}({d}_{T}-1)X, & if\,s(i)=TC\,({\rm{trustworthy}}\,{\rm{consumer}}),\\ {k}_{TP}Temp+{k}_{UP}({d}_{U}-1)X, & if\,s(i)=UC\,({\rm{untrustworthy}}\,{\rm{consumer}}).\end{array}$$

As sharing economy platforms normally would provide their providing users with some sort of penalty or insurance (e.g., for damages to providers’ assets or when consumers are left stranded), which mitigates a substantial amount of the costs for interacting with untrustworthy counterparts, in this study we model *X* and *S* to be smaller than *R*. That is:

2 $$2\cdot R > Temp > R > S > X.$$

We are also interested in the global net wealth of the population, *W*, calculated as $W={\sum }_{i=1}^{Z}\,{w}_{i}$. By running the model for a maximum number of time steps in a synchronous manner, at each time step *t* all agents in the population decide on which strategies to choose based on the population state of the previous time step (i.e., *t* − 1). This means that the actions of others at time step *t* will not affect the focal agent’s decision during the same time step^17^.

### Strategy update

The strategy of each agent, *s*(*i*), can change during the game, as each agent is given an opportunity to update its strategy through an evolutionary update process. We may interpret this activity of strategy update as information exchange in a social learning process, where agents in the population imitate the strategies of others^38^. Strategy imitation occurs in all time steps during the game. At time step *t*, a focal agent *i* (independent from its strategy) evaluates its previous payoff in *t* − 1 and decides whether to imitate a neighbouring (connected) agent’s strategy or not by applying an evolutionary update rule. Update rules of imitative nature represent a situation where bounded rationality or lack of information forces players to copy (imitate) others’ strategies^40^. These update rules are widely employed in the relevant literature to model evolutionary dynamics.

For this work, we use a proportional imitation rule (also known as the replicator dynamics)^20^, which is pairwise and stochastic, similar to some of the recently studied evolutionary imitation schemes^41^. The proportional imitation rule is also similar to Fermi’s rule^42^, except it allows for the possibility of making mistakes when imitating (i.e., players can imitate others who are gaining less). Proportional imitation has been chosen here because it was previously used in other evolutionary trust games^13,26^. Under this rule, an agent *i* may adopt one of the four possible strategies for the game (i.e., $s(i)=\{TP,UP,TC,UC\}$) from another agent *j* that is interacting with *i* at time step *t*. Specifically, the update rule works as follows. Let us denote a randomly selected neighbour of focal agent *i* as *j*. The rule first evaluates if the individual payoff value of *j* in the previous time step, ${w}_{j}^{t-1}$, is higher than that of the focal agent (${w}_{i}^{t-1}$). If it is higher, agent *i* will adopt the strategy of agent *j*, *s*(*j*), by a probability that depends on the difference between their payoffs:

3 $$pro{b}_{s(i)}^{t}\,s(j)=\frac{{\rm{\max }}\,\mathrm{\{0},{w}_{j}^{t-1}-{w}_{i}^{t-1}\}}{\phi },$$

where *ϕ* is the difference of maximum and minimum possible individual net wealth between two arbitrary agents at time step *t* − 1 to have $pro{b}_{s(i)}^{t}s(j)\in [0,1]$.

## Results

We carried out simulation experiments to analyse evolutionary dynamics of the population and effects of different initial configurations, punishment for untrustworthy providers, and insurance for consumers. We first describe the experimental setup, and then discuss the simulation results in three main sub-sections.

### Experimental setup

In our experiments, all agents in the population were placed on a real social network^43^. This network was built from the email traffic at Rovira i Virgili University in Spain: each email address is considered as a node, and two nodes are linked if there is an email exchange between them. In total, there are 1,133 nodes, resulting in a population of 1,133 agents (i.e., *Z* = 1,133). The network’s clustering coefficient is 0.254, and its average shortest path length is 3.606, having clear communities within the network (see^43^ for more details).

We set the simulations to 5,000 time steps, and all of them were repeated for 50 independent Monte Carlo runs. Reported results were calculated by averaging the last 1,250 time steps (i.e., the last 25%) of each run. In every time step, each agent plays the game iteratively with other directly-connected agents and decides whether to change its own strategy. Payoffs of the agents were calculated based on the payoff matrix in Table 1.

### Analysing different initial conditions

In this section, we report on the results for different values of *R* and *X*, covering different trust situations ranging from the game being *easier*, *moderate*, and *harder*. We set *S* = 20 and *Temp* = 40, with *R* ranging from 21 to 32 and *X* from 1 to 19. These ranges have been chosen so that all possibilities with *R* and *X* are covered, without violating the constraint of Eq. 2. Here, we did not consider punishment and insurance (i.e., $p={d}_{T}={d}_{U}=0$). We also did not include *R* values >32 and <40, because in our preliminary study^27^ we found that varying *R* between 32 and 40 does not change the evolutionary outcomes (i.e., results of $32 < R < 40$ are identical to that of *R* = 32). Five scenarios with different initial distributions of the population strategies–*TP*, *UP*, *TC* and *UC*–were considered. The five scenarios are as follows:

- Scenario 1: the four strategies are divided proportionally ($TP\approx UP\approx TC\approx UC$).
- Scenario 2: more consumers than providers ($TP\approx UP\approx 0.05\cdot Z$ and $TC\approx UC\approx 0.45\cdot Z$).
- Scenario 3: more providers than consumers ($TP\approx UP\approx 0.45\cdot Z$ and $TC\approx UC\approx 0.05\cdot Z$).
- Scenario 4: more untrustworthy than trustworthy players ($TP\approx TC\approx 0.05\cdot Z$ and $UP\approx UC\approx 0.45\cdot Z$).
- Scenario 5: more trustworthy than untrustworthy players ($TP\approx TC\approx 0.45\cdot Z$ and $UP\approx UC\approx 0.05\cdot Z$).

**Figure 1:**
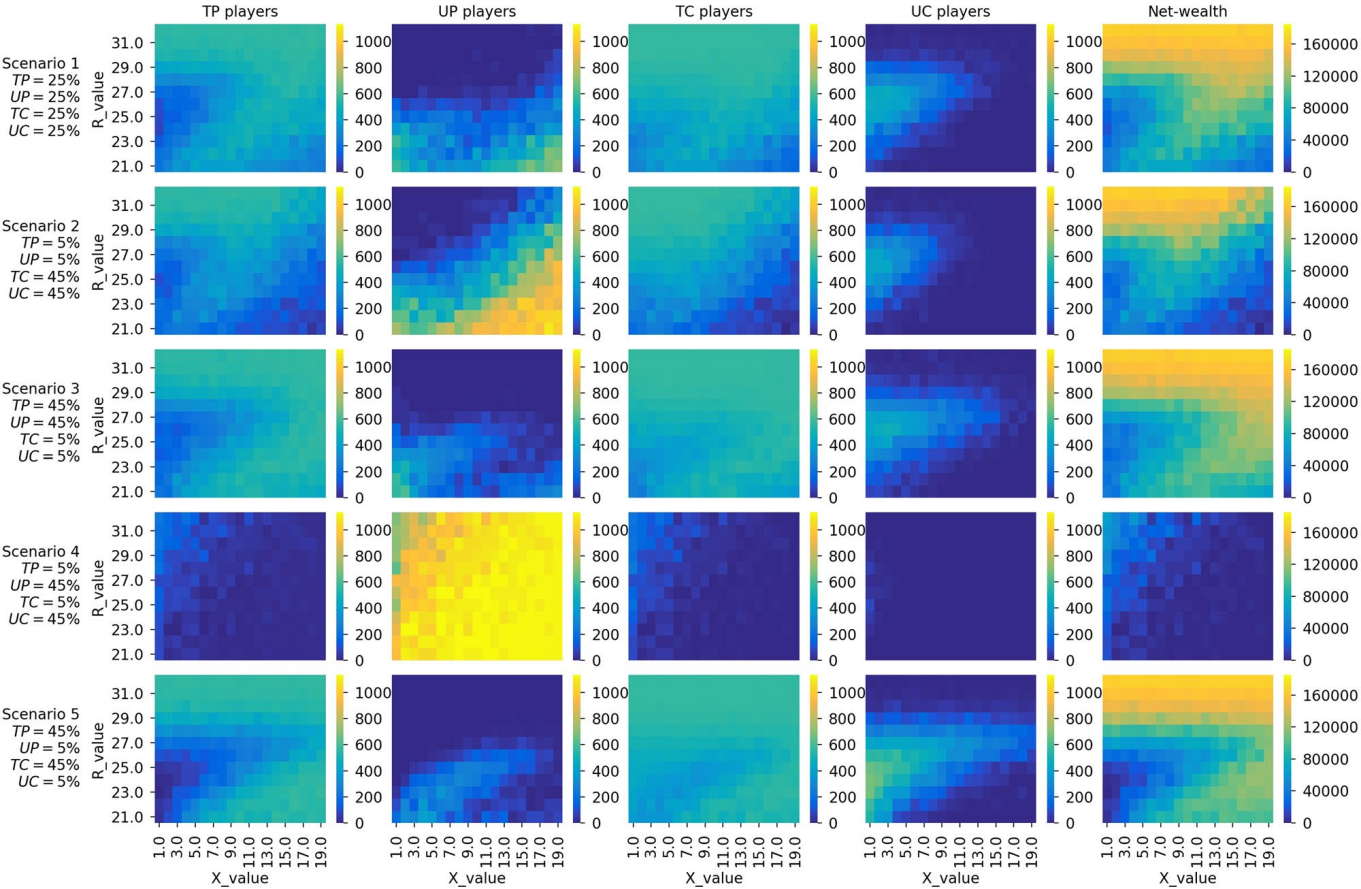
Heatmaps showing the final state of the populations (the number of players having strategies *TP*, *UP*, *TC*, and *UC*, $\in \mathrm{[0,1133]}$) and net wealth for different values of *R* and *X* on 5 different initial population configurations, with *S* = 20 and $Temp=40$.

Figure 1 shows heatmaps of sensitivity analysis on the two parameters *X* and *R* for the five scenarios. Heatmaps in the first column show the average numbers of *TP* players in the last quartile of the simulation (i.e., the last 1,250 time steps). Heatmaps in the second, third, and fourth columns present the same average numbers but for *UP*, *TC*, and *UC* players. Finally, the last column shows the average global net wealth in the last quartile.

We can see from the heatmaps of Fig. 1 that the first and fifth scenarios are the best in terms of promoting trust in the sharing economy. These two scenarios have at least 50% of the initial population as trustworthy individuals, either providers or consumers. Trust is also easy to form when the initial population has the same number of trustworthy and untrustworthy players but with a majority of them providers (i.e., Scenario 3). Scenario 4, where only 10% of the initial population are trustworthy, is a scenario with low levels of trust and global net wealth, even when the *R* values are high (top cells of each heatmap).

Another observation we can make from the heatmaps is that there is a parameter range where promoting trust is particularly complicated. Within this parameter range, specifically when $R\,\lessapprox \,28$ and $X\,\lessapprox \,10$, *UC* players are dominant. It is worth noting that this is the only parameter space where *UC* players are observed to be dominant. For the rest of the configurations, they are almost completely wiped out.

**Figure 2:**
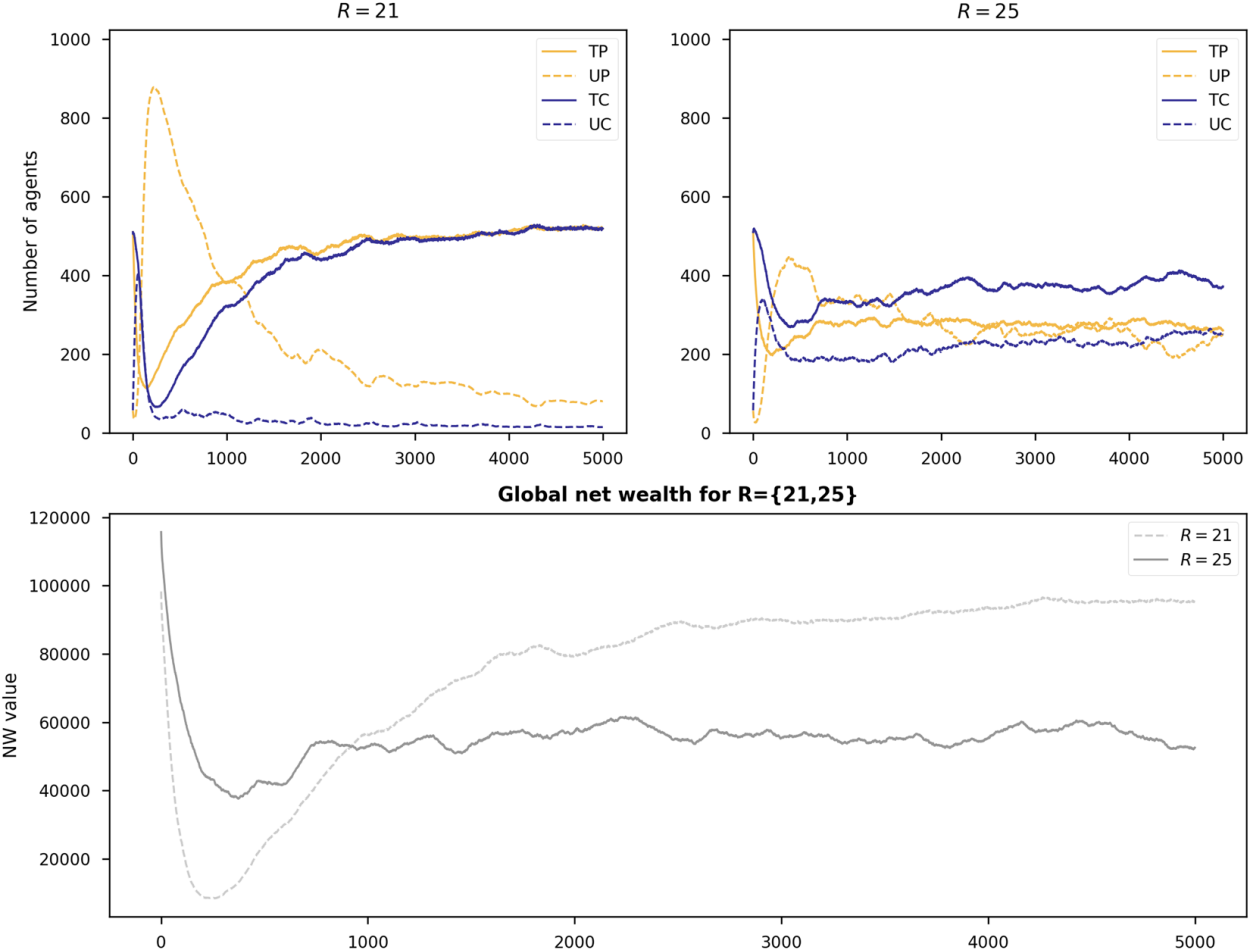
Time series evolution of the strategies and net wealth for Scenario 5 (90% of the initial population are trustworthy players) when *X* = 10 and *R* = {21, 25}.

The most interesting observation, however, is that for some values of *X*, decreasing the reward value for being either a trustworthy provider or consumer (*R*) facilitates trust in the environment. This behaviour can be seen as a vortex at $X\in \mathrm{[5,\; 13],}R\in \mathrm{[21,\; 28]}$ in the heatmaps of Scenarios 1, 3, and 5. To understand this behaviour better, we show in Fig. 2 the time series evolution of strategies and net wealth for *R* = {21, 25} and *X* = 10 with Scenario 5, where 90% of the players are initialised as either *TP* or *UP*. This plot explains why, in this vortex area, when increasing *R* (higher payoffs to trustworthy players) the global net wealth decreases. As we can see, there is a peak for *UP* (purple line) at the beginning of the simulation when *R* = 21. This, in turn, eliminates *UC* players (orange line) to a large extent. Without *UC* players, *TP* and *TC* players (red and green lines) are ‘revived’. As the numbers of *TP* and *TC* players increase, the number of *UP* players decreases dramatically. Moving our attention to the case of *R* = 25, we see that there is a lack of *UP* players at the beginning of the simulation. This allows *UC* to exploit *TP*, thereby diminishing the dominance of the latter. This behaviour is reflected in the bottom plot of Fig. 2, where the net wealth rebounds for *R* = 21 after 200 time steps, surpassing the net wealth obtained under the setting of *R* = 25 (*a priori*, a better configuration to promote trust). We attribute this to *TP* and *TC* players ‘working’ hand-in-hand to help each other out under difficult circumstances.

### Influence of Penalties on Untrustworthy Providers

**Figure 3:**
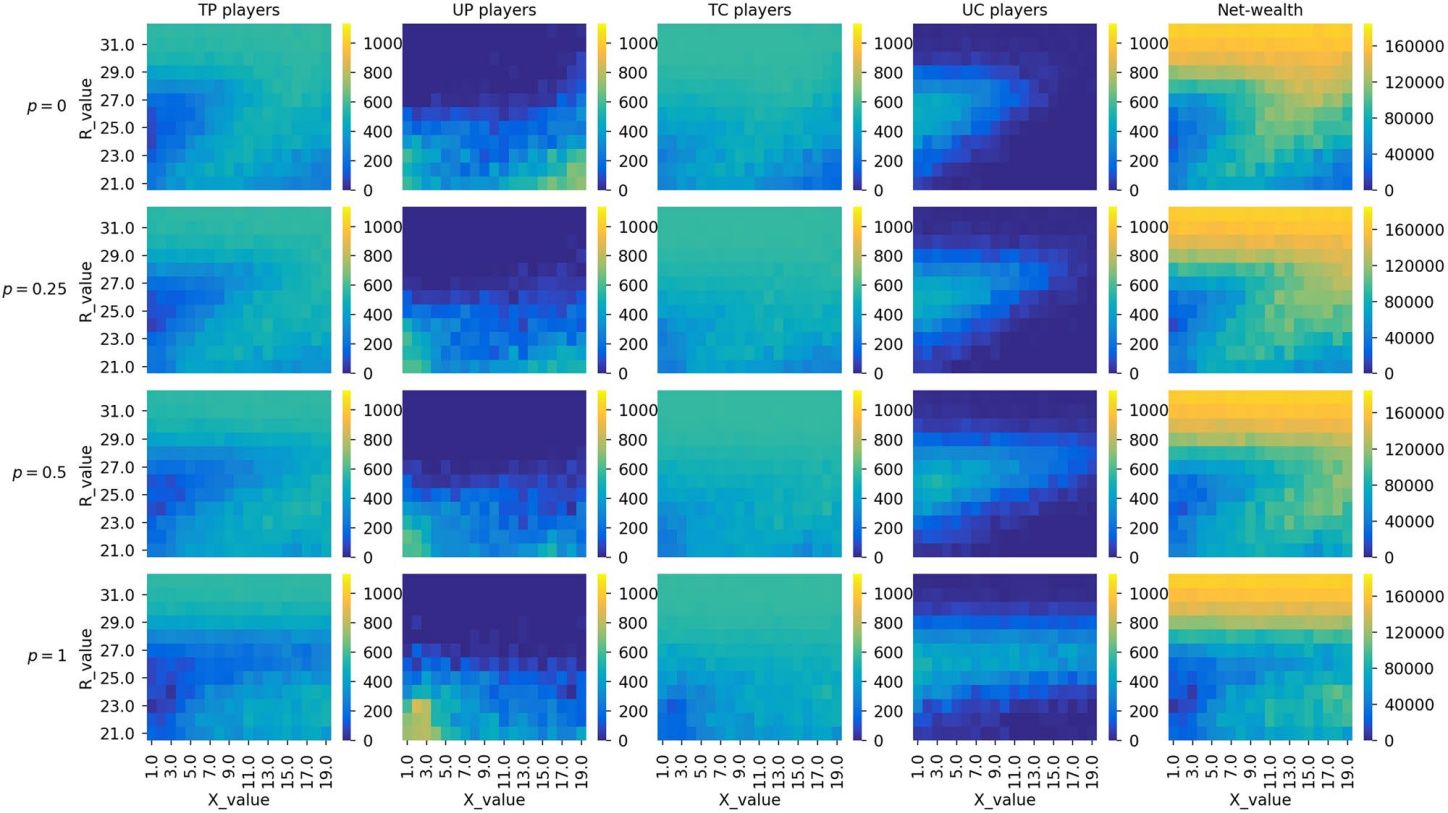
Heatmaps showing the final state of the populations (the number of players having strategies *TP*, *UP*, *TC*, and *UC*, $\in \mathrm{[0,1133]}$) and net wealth for different values of *R* and *X* when setting penalty *p* to $\mathrm{0,0.25,0.5}$, and 1. Simulations were run with the initial population having 25% of each strategy type, and *S* = 20 and $Temp=40$ (i.e., Scenario 1).

In this section, we report on how penalties applied to untrustworthy providers *UP* influence the dynamics of the game. We set the initial population to have 25% of each strategy type. Figure 3 plots the heatmaps of sensitivity analysis on *X* and *R* as done in the previous section, but this time with different values of penalty parameter *p* of the model. Here, heatmaps in the first row show the model dynamics without penalisation (same as Scenario 1 in the previous section). The subsequent rows show the final states of the population and net wealth in the last quartile of the simulation for *p* equal to 0.25, 0.5, and 1, respectively.

By looking at the plots, we can see that increasing the value of penalties for *UP* has two major effects. The first one is positive for promoting trust, because it reduces the number of *UP* around the bottom right area of the plots (i.e., $X\,\gtrapprox \,10$ and $R\,\lessapprox \,25$). The second one is negative for trust in the sharing economy, as penalties lead to the decrease of trustworthy players and an increase in *UP* and *UC* when *R*’s values are moderately high and *X* is around its lowest limit.

**Figure 4:**
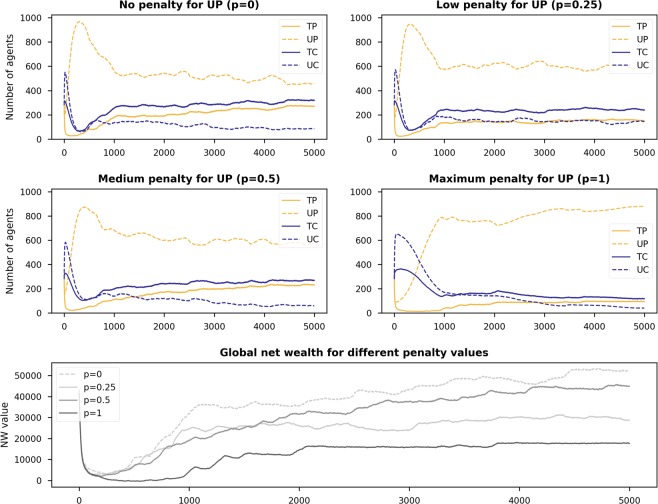
Time series evolution of the strategies and net wealth for different penalty values ($p=0,0.25,0.5$, and (1) when *X* = 2 and *R* = 22.
- Point $(X=13,R=26)$ (evolution in Fig. 5): We again see that increasing the penalties against *UP* is not positive for promoting trust in the game. If there is no penalty, the population is dominated by trustworthy players (see the first plot in Fig. 5). With an increase in the penalty values, however, the number of trustworthy providers and thus net wealth decreases. The main difference between this $(X=13,R=26)$ and the previous point $(X=2,R=22)$ is that penalties are able to reduce the number of *UP* players in the population but without much positive impact on the global net wealth.

To explain the reasons behind these dynamics, we plot the time series evolution of three points located around the main areas where the changes occurred:

- Point $(X=2,R=22)$ (evolution in Fig. 4): Here, *UP* players are more dominant when penalties against them are increased, and consequently, there is a fall in the global net wealth within the population. This observation, however, is counter-intuitive. If we look at the plots in Fig. 4, we see that at the very beginning of the simulation *UP* players are not doing well, and both types of consumers, untrustworthy ones in particular, appear to be more dominant. As the population is dominated by *UC*, *TP* is exploited giving rise to *UP* again.

**Figure 5:**
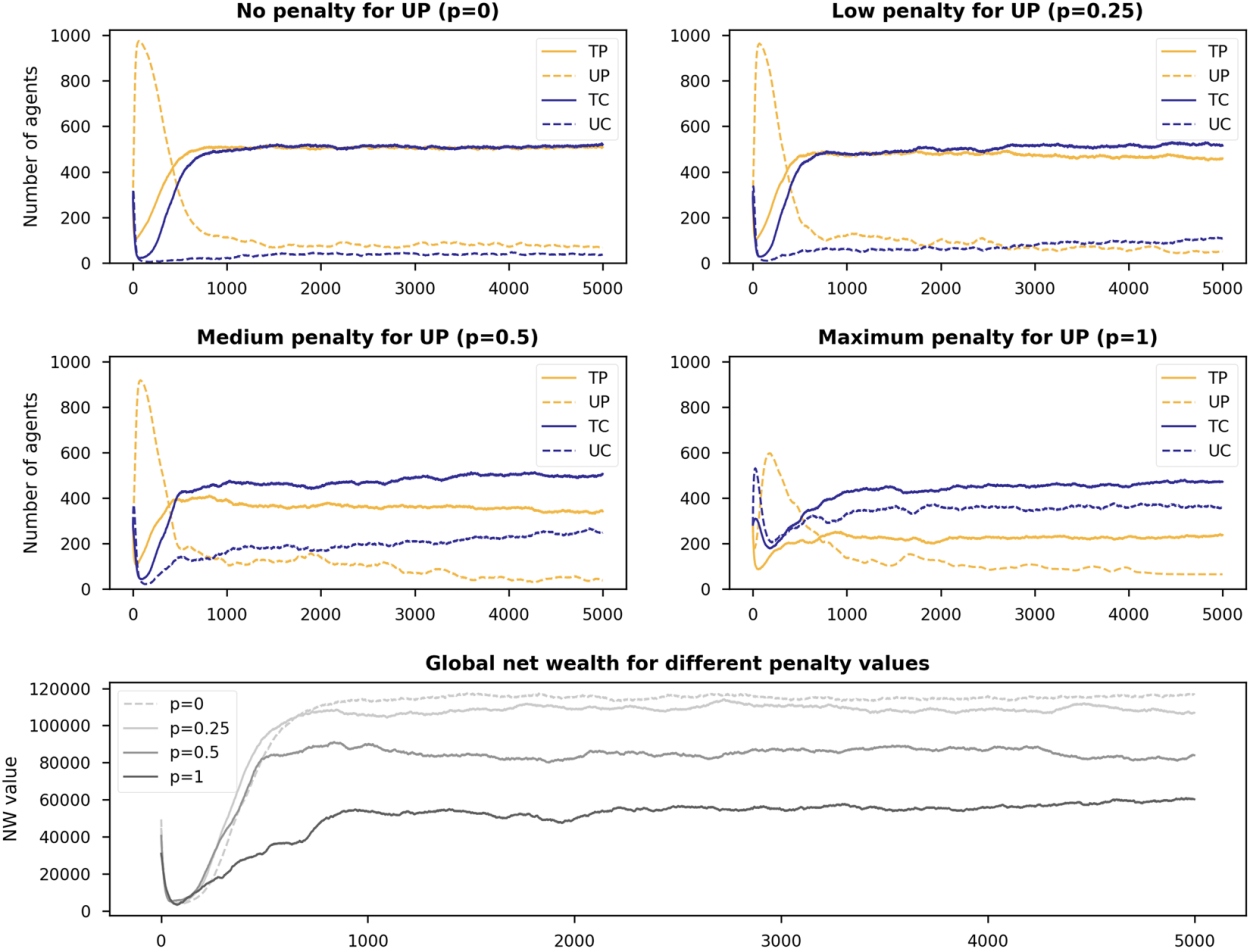
Time series evolution of the strategies and net wealth for different penalty values ($p=0,0.25,0.5$, and (1) when *X* = 13 and *R* = 26.
- Point $(X=19,R=22)$ (evolution in Fig. 6): This point lies in an area where using penalties against *UP* players is positive for promoting trust. As we can see in the net wealth plot of Fig. 6, the best strategy here is to set *p* to 1 (its maximum value). When the reward is low (*R* = 22) and the ‘deposit’ consumers pay after initiating a transaction is high (*X* = 19), penalising *UP* increases the net wealth as the number of *UP* players is greatly reduced in the population.

**Figure 6:**
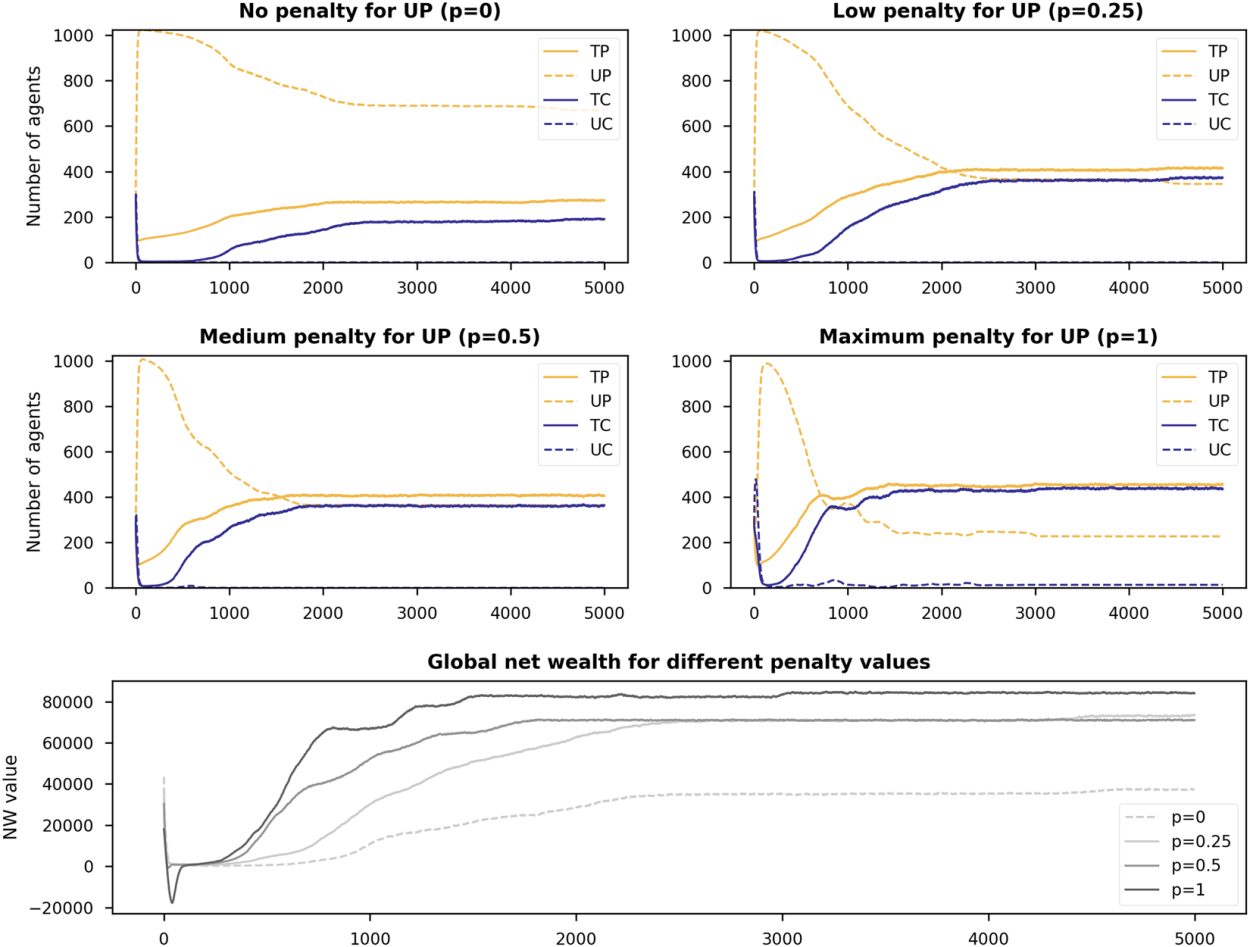
Time series evolution of the strategies and net wealth for different penalty values ($p=0,0.25,0.5$, and (1) when *X* = 19 and *R* = 22.

Summing up, we have observed that for the majority of *X* and *R* values, having a high penalty value for *UP* players does not increase net wealth, as it does not necessarily lead to the elimination of untrustworthiness from the population. For instance, in cases with both low *X* and *R* values, the number of *UP* players, surprisingly, can be higher when increasing the penalty value.

### Influence of having insurance for consumers

In this section, we report on the influence of having insurance for consumers in the case of providers being untrustworthy. We examined two possibilities. The first was to have insurance only for trustworthy consumers *TC* (parameter *d*_*T*_), while in the second we applied the same insurance to both types of consumers *TC* and *UC* (*d*_*T*_ and *d*_*U*_ with *d*_*T*_ = *d*_*U*_), in order to understand the game dynamics when insurance is in place for all the consumers regardless of their trustworthiness. Again, we set the initial population to be 25% for each type of strategy. For the experiments reported in this section, we did not consider penalties for *UP* (i.e., *p* = 0) to make it easier for us to analyse the results.

**Figure 7:**
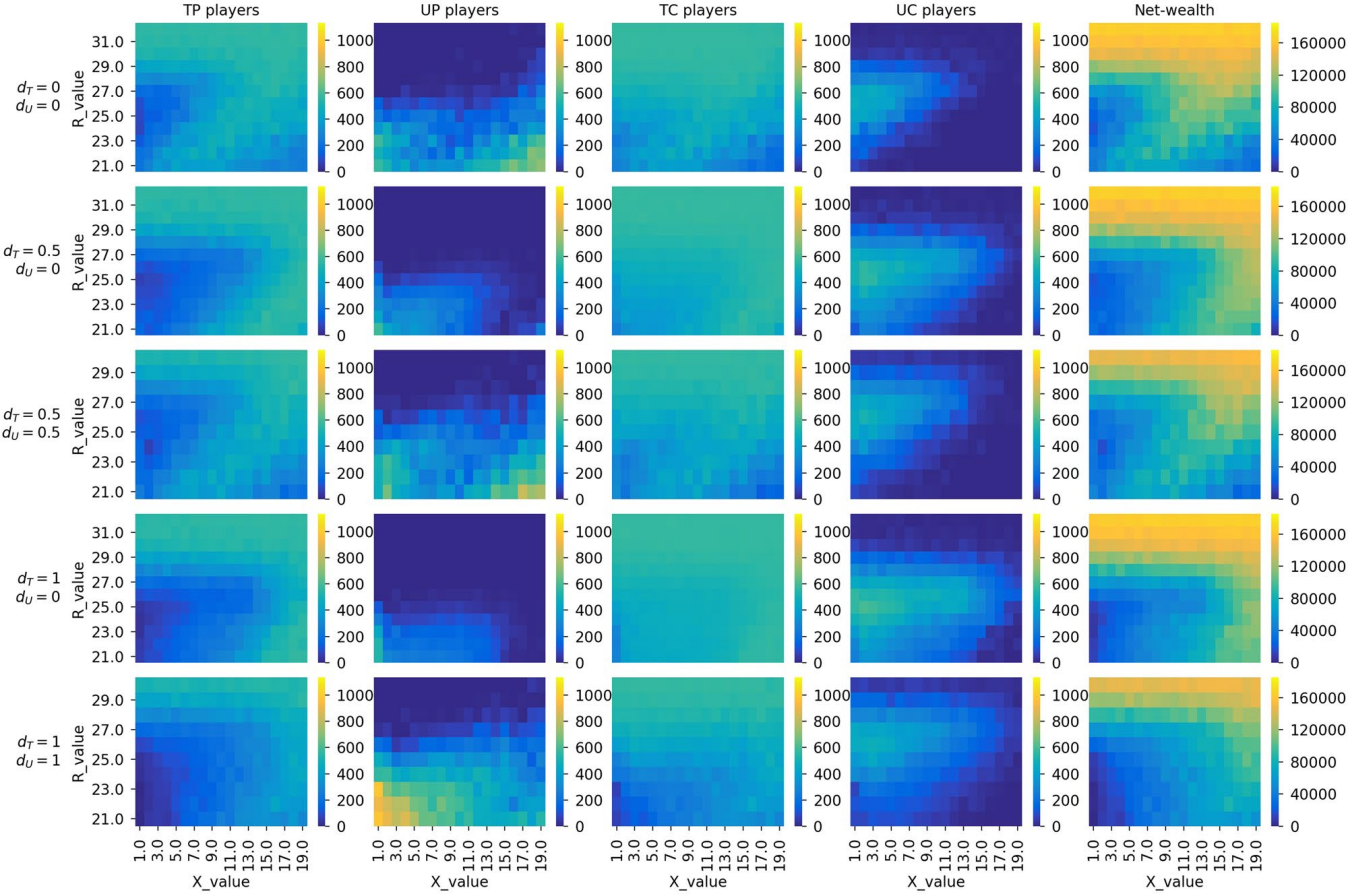
Heatmaps showing the final state of the populations (the number of players having strategies *TP*, *UP*, *TC*, and *UC*, $\in \mathrm{[0,1133]}$) and net wealth for different values of *R* and *X* when having insurance in place (${d}_{T}$, ${d}_{U}$ equal to $\mathrm{\{0,0\},\{0.5,0\},\{0.5,0.5\},\{1,0\}}$, and {1, 1}) for *TC* and *UC* players. Simulations were run with the initial population having 25% of each strategy type, and *S* = 20 and $Temp=40$ (i.e., Scenario 1).

Heatmaps in Fig. 7 show the final state of the population and net wealth with no insurance (the first row) and also with varying values of *d*_*T*_ and *d*_*U*_ (five combinations of values $\{0,0\},\{0.5,0\},\{0.5,0.5\},\{1,0\}$, and {1, 1}). Upon close inspection of the five rows of heatmaps, we see that:

- In general, there are more consumers in the final population (both *TC* and *UC*) when *d*_*T*_ is in place. This consequently reduces *UP* and *TP* in the population. Although the reduction of *UP* appears to be good for the sharing economy, reduction in the number of *TP* changes the net-wealth landscape (see the last column of heatmaps) by shifting high net wealth values (yellowish cells) towards the bottom right corner. Insurance for *TC* is therefore particularly good with higher *X* values.

- *UP* players almost completely disappear from the final population when we have insurance for just *TC* (i.e, *d*_*T*_ > 0 and *d*_*U*_ = 0). In fact, having insurance in place for the consumers seems more appropriate for reducing the number of *UP* than imposing penalties on them.
- The use of insurance to protect *UC* through *d*_*U*_ moves players towards untrustworthy strategies (*UP* and *UP*). As being a *UP* player is always safe, trustworthy players would move to become an *UP*, resulting in low net wealth values for almost all pairs of *X*/*R* configurations.

The main finding here is that insurance is preferable for just *TC* (*d*_*T*_), if we want to increase the net wealth. Ideally, insurance should not be applied to *UC* (i.e., *d*_*U*_ = 0). Similar to the case of having penalties for *UP*, the impact of using *d*_*T*_ is preferable with high *X* values and low *R* values.

## Discussion

In this work, we presented an evolutionary trust game to investigate the formation of trust in the sharing economy with a connected population of players. The importance of trust in this context is clear, as the trusting relationships involved here are complex. Our sharing economy trust model consists of a finite set of agents occupying the nodes of a real network, and the edges denote ‘transactions’ between them. Every agent can choose between being a trustworthy provider, an untrustworthy provider, a trustworthy consumer, or an untrustworthy consumer. The proposed game model includes mechanisms to punish untrustworthy behaviours and protect consumers by means of penalty and insurance, respectively.

Systematic computational experiments across a range of *R* and *X* values and different initial population distributions showed that trust can be formed when the reward values are high and/or *X* values are high, except if the initial population has limited trustworthy players (e.g., only 10% of them). The simulation results also uncovered a surprising phenomenon, i.e., untrustworthy consumers are almost never dominant. Detailed analysis revealed that even though untrustworthy consumers can exploit trustworthy providers, they are ‘vulnerable’ to untrustworthy providers. On the contrary, trustworthy providers and consumers ‘cooperate’ with each other. This points to the importance of a balancing effect of trustworthiness due to the mutual trust constellation. More specifically, untrustworthiness of the consumers (e.g., causing financial and/or psychological costs to the provider due to theft or damage) is retaliated against with untrustworthy behaviour by the providers (e.g., deviating from the agreed-upon level of access to the asset), driving consumers to behave trustworthily. This effect also appears as a vortex in the parameter space, showing that increasing the reward value *R* is sometimes worse for the spread of trustworthiness.

Our experiments also extended to studying the influence of penalties for untrustworthy providers and insurance for consumers. We observed that for the majority of the tested *X* and *R* configurations, having high penalty values for *UP* players does not increase net wealth, and more importantly, in cases with both low *X* and *R* values, the number of *UP* players is surprisingly higher when the penalty value is increased. In terms of insurance for consumers, we found that it is preferable to only protect trustworthy consumers through parameter *d*_*T*_ if we want to increase net wealth. As with the case of having penalties in place, the benefit of applying insurance for *TC* is more obvious with high *X* and low *R* values.

Our future work will involve investigating the model dynamics using different network topologies (e.g., see^44,45^), which impact information diffusion differently. Other important avenues for possible future research include the use of different update rules and temporal networks that evolve the connections of players during the simulation.
